# Association of anthropometric parameters with amplitude and crosstalk of mechanomyographic signals during forearm flexion, pronation and supination torque tasks

**DOI:** 10.1038/s41598-019-52536-4

**Published:** 2019-11-07

**Authors:** Irsa Talib, Kenneth Sundaraj, Chee Kiang Lam

**Affiliations:** 1 School of Mechatronic Engineering, Universiti Malaysia Perlis (UniMAP), Arau, 02600 Perlis Malaysia; 2 Centre for Telecommunication Research & Innovation, Fakulti Kejuruteraan Elektronik & Kejuruteraan Komputer, Universiti Teknikal Malaysia Melaka (UTeM), Durian Tunggal, 76100 Melaka Malaysia

**Keywords:** Neurophysiology, Skeletal muscle

## Abstract

This study aimed to quantify the association of four anthropometric parameters of the human arm, namely, the arm circumference (CA), arm length (LA), skinfold thickness (ST) and inter-sensor distance (ISD), with amplitude (RMS) and crosstalk (CT) of mechanomyography (MMG) signals. Twenty-five young, healthy, male participants were recruited to perform forearm flexion, pronation and supination torque tasks. Three accelerometers were employed to record the MMG signals from the biceps brachii (BB), brachialis (BRA) and brachioradialis (BRD) at 80% maximal voluntary contraction (MVC). Signal RMS was used to quantify the amplitude of the MMG signals from a muscle, and cross-correlation coefficients were used to quantify the magnitude of the CT among muscle pairs (BB & BRA, BRA & BRD, and BB & BRD). For all investigated muscles and pairs, RMS and CT showed negligible to low negative correlations with CA, LA and ISD (*r* = −0.0001–−0.4611), and negligible to moderate positive correlations with ST (*r* = 0.004–0.511). However, almost all of these correlations were statistically insignificant (*p* > 0.05). These findings suggest that RMS and CT values for the elbow flexor muscles recorded and quantified using accelerometers appear invariant to anthropometric parameters.

## Introduction

Mechanomyography (MMG) is a widely used myographic technique that has a wide range of clinical implications^1^. Although easy to use, subjective factors, such as anthropometric parameters, recording protocols, and environmental conditions, affect the acquired myographic signals and casts doubts on the deductions made from physiological observations obtained using these techniques^2^. MMG has been used for the assessment of skeletal muscle activity over the last three decades^3^. This technique provides more global information on muscle function as precise placement of the sensor over the muscle is not critical in MMG unlike in surface electromyography (sEMG)^4^. MMG is a suitable alternative to sEMG, specifically for the assessment of muscle fatigue^5,6^ and the study of muscle contractile properties^7^. Other benefits of MMG include its ease in operation^8^, the stability of the signal components and the negligible effect of skin impedance on the signal^9,10^.

Despite these advantages of MMG, crosstalk (CT) is a drawback that hinders the use of MMG in clinical applications^11^, the development of prosthetic control^12,13^, observations of muscle function^14–17^, the monitoring of the motor unit activity^18–20^ and observations on neuromuscular blockade^21^. CT can be defined as the contamination of the target muscle signals by the signals from muscles adjacent to the target muscle^22^. Theoretically, MMG signals from two separate muscles should not have high level of % common signal as these signals are from two unique sources and it is less probable for these signals to have identical shape of waveform. Thus, CT constitutes a major problem for the clinical applications of myographic signals^2^, but proper precautionary measures might diminish these effects.

Although it has been found that the anthropometric parameters of humans influence the amplitude and CT of myographic signals^2^, the link between these parameters and the amplitude of the MMG signal from a muscle or CT due to adjacent muscles remains to be fully understood, particularly those associated with the elbow flexor muscles. To the best of our knowledge, the current literature highlights four anthropometric parameters of the human arm that have been found to influence the amplitude and CT of myographic signals: the arm circumference(CA), the arm length (LA), the skinfold thickness (ST) and the inter-sensor distance (ISD).

The circumference and length of a limb might reflect the size and volume of its muscles^23^. A larger muscle produces a myographic signal with a greater amplitude^2^. Only one previous study^24^ has quantified the correlation between the CA and RMS. The authors found a weak negative correlation between these parameters measured from the biceps brachii (BB) using an accelerometer during isometric contractions. The correlation between the forearm circumference and CT in MMG signals was quantified by^25^ during an isometric grip force task and was found to be weakly positive. Similarly, the effect of variations in the forearm circumference on CT in sEMG signals was also discussed by^26^. These researchers recorded the sEMG signals during the forearm pronation, supination and neutral posture while exerting a low grip strength. The researchers observed that the individuals with larger forearms showed lower CT values. The correlation between the forearm length and CT in MMG signals was quantified by^25^ during the isometric grip force task and was found to be weakly negative.

The ST could be a major issue in some clinical applications of myographic signals^2^ because it acts as a low-pass filter that tends to attenuate the signal amplitude^27^. The effect of variations in the ST on the RMS from the quadriceps muscle has been assessed in a few studies^28–33^. Among these studies, only those conducted by^28–30^ quantified the correlation between the ST and RMS, and they observed a negative correlation between these two parameters for the quadriceps. The effect of variations in the ST on RMS from the BB has only been assessed in one previous study^24^, and these researchers also quantified the correlation between the ST and RMS and found a weak positive correlation between the two parameters during maximal voluntary contraction. The effect of variations in the ST on CT in sEMG signals has only been assessed by^34–36^, and it was found that CT in sEMG signals increased with increases in the ST.

The proximity of muscles also influences CT in myographic signals^2^. The radial distance between the sensors placed over the muscles’ belly is called the inter-sensor distance (ISD) of the associated muscle pairs, and this variable has been found to be representative of the inter-muscular distance. Thus, a correlation of the ISD with CT might clarify the effect of proximity between muscles on the MMG signal. The CT in the MMG signals from forearm muscles (69%) recorded by^37^ was higher than that in the MMG signals from the quadriceps (51%) recorded by^38^, and this finding could be due to the closer proximity among the forearm muscles compared with that among the quadriceps muscles. The authors in^39^ found that the CT in sEMG signals from wrist-dedicated flexors was greater than that in the signals from digit-dedicated flexors due to the smaller ISD in wrist-dedicated flexors compared with that in digit-dedicated flexors.

Based on all the observations on the four anthropometric parameters of interest obtained in previous myographic studies, the following deductions can be made. First, none of the previous myographic studies using MMG or sEMG have quantified a correlation between the ST and CT. Although^35,36^ made some observations on the effect of the ST on CT in sEMG signals, both the studies were based on a model that has not been verified using an *in vivo* experimental protocol. Second, none of the previous MMG studies have observed the effect of the ST on CT. Third, none of the previous MMG studies considered forearm pronation or supination tasks and elbow flexor muscles to observe the effect of anthropometric parameters on RMS and CT. Fourth, the correlation of four anthropometric parameters with RMS and CT has not yet been quantified for three elbow flexors working in synergy.

These research gaps appear crucial because variations in anthropometric parameters might alter the RMS and/or CT, which could challenge the design of MMG-based prosthetic control or observations on neuromuscular blockade^21^. The deductions on muscle function and motor unit activity from MMG signals might be altered due to these effects. Therefore, it is important to analyse the correlation of anthropometric parameters with RMS and CT to obtain a precise definition of its usage as a muscle assessment tool. Based on the observations from literature, the null hypothesis for current study states that there is no association between MMG parameters and anthropometric variables. To test the hypothesis, we investigated the correlation of ST, CA, LA and ISD with the RMS and CT of MMG signals from three elbow flexor muscles – the BB, brachialis (BRA) and brachioradialis (BRD). The intermediate size, the inter-muscular distance and the accessible locations of the muscles, makes these elbow flexors an optimal choice for the quantification and analysis of CT in MMG signals^40^. All three muscles are elbow flexors, but the BB and BRD also serve as a forearm supinator and a forearm pronator, respectively^41^

## Methods

### Subjects

Twenty-five young, healthy and untrained male subjects [(mean(SD): age = 25.52(4.54) years, weight = 68.21(9.65) kg, height = 167.52(3.17) cm] with no history of neuromuscular injury participated in the experiment. The anthropometric measures of the subjects recruited in this experiment cover broad ranges of the weight, height and LA of the global population comprising different races, as discussed by^42^. All the subjects belonged to second and third quartiles of BMI excluding malnutrition and obese subjects^43^. The study was approved by the local Medical Research & Ethics Committee (MREC), Ministry of Health, Malaysia (NMRR-14-822-22001) and adhered to the guidelines established by the Declaration of Helsinki.

### Measurement of anthropometric parameters

The personal details were taken, and a written informed consent was obtained from the subjects during the familiarization session. The anthropometric measurements of the subjects were recorded during both the familiarization session and experimental procedure with a gap of 24–36 hours. The tester who recorded the measurements in our study had performed a priori more than 150 measurements of anthropometric parameters. Only after the tester had enough perfection in taking these measurements (confirmed by a physician present on site throughout the experiment), were the actual measurements taken. The measurement from the glenohumeral joint and olecranon was considered the LA. The CA was measured from proximal and distal to the shoulder and middle of the arm while the elbow joint was flexed at 90°. The mean of these three measured values was considered the CA. The location at which the sensor was placed over each muscle was marked and verified by a medical doctor. The ISD was measured between two associated muscles using these marked locations. The ST was measured using a digital skin-fold thickness analyser (ABS digital body fat calliper, range = 0–50 mm, resolution = 0.1 mm, accuracy = ±0.2 mm, Shanghai ENOVO Industrial Ltd., China) over the marked locations of the sensors on each muscle. The mean value of the ST between two associated muscles was used for the CT analysis. The details of anthropometric parameters of all the subjects are given in Table 1.

**Table 1:** Details of the anthropometric parameters of all the subjects.

Subject #	LA (cm)	CA (cm)	ST BB (mm)	ST BRA (mm)	ST BRD (mm)	ISD P1 (cm)	ISD P2 (cm)	ISD P3 (cm)
1	35.5	34.5	13.1	15.6	12.8	6.5	6.0	13.0
2	36.0	29.8	10.4	14.4	14.4	5.5	9.5	12.0
3	36.5	32.8	8.4	10.0	9.6	7.5	6.0	11.5
4	41.0	32.8	10.8	14.4	14.8	9.5	7.5	15.5
5	35.0	28.5	8.8	9.2	8.8	7.5	7.0	13.0
6	38.0	35.0	8.0	10.4	10.0	7.5	7.5	13.0
7	37.0	26.7	10.4	11.2	10.25	6.5	7.5	12.5
8	35.5	28.3	10.4	8.1	10.4	6.5	7.5	13.0
9	35.0	32.3	7.6	9.6	10.8	7.0	7.0	13.0
10	36.0	35.5	8.0	9.2	8.8	9.0	6.5	14.5
11	39.5	35.1	10.4	8.8	8.8	8.0	7.5	14.0
12	38.0	31.6	8.4	10.8	10.4	8.5	8.5	15.5
13	38.5	34.3	7.6	9.2	10.0	8.5	8.5	15.0
14	36.5	35.5	11.6	12.8	13.2	9.0	7.0	15.0
15	33.5	33.8	6.8	10.0	8.4	7.5	8.0	15.0
16	35.0	30.0	7.6	8.8	8.8	7.5	8.0	14.5
17	33.0	24.8	7.2	6.8	7.2	7.5	6.5	13.0
18	37.0	33.0	8.3	8.0	7.4	6.5	8.0	13.5
19	37.0	30.1	5.2	7.6	8.0	10	8.0	16.0
20	37.5	34.5	8.0	10.4	9.6	8.5	9.0	16.5
21	36.0	31.6	7.2	8.8	8.4	7.5	7.5	13.5
22	36.0	32.6	8.0	8.4	9.6	8.0	8.0	14.5
23	39.0	39.8	8.4	9.2	11.2	8.0	8.5	15.5
24	36.5	28.5	6.4	8.2	7.6	8.0	7.0	14.5
25	40.5	33.5	8.4	12.4	14.4	9.0	8.5	16.5

### Experimental procedure

In the first session, called familiarization session, the subject was asked to perform a set of warmup exercises, which included arm stretching, elbow joint flexion and five to six repetitions of biceps curls with low weights. At the end of the warmup session, the subject was ready for MVC determination. MVCs were then determined for the isometric forearm flexion, supination and pronation tasks through three trials with a 2 minutes rest period between any two consecutive trials and a 10 minutes rest period between any two consecutive tasks. During each task, the subject sat comfortably on a chair with a straight back, shoulders slightly abducted and 90° angle between arm and forearm. The subject’s wrist flexion was restricted using a wrist-hand orthosis and the angle between the subject’s arm and forearm was ensured using a digital goniometer. The subjects’ arm was kept parallel to the body torso and shoulder flexion/extension was visually maintained at this posture by a dedicated observer present parallel to sagittal plane of the subject. The shoulder adduction angle (empirically determined a priori to be about 3° using an electronic goniometer) was such that the subject’s arm did not touch the body it was visually maintained by another dedicated observer present parallel to the frontal plane of the subject. Both independent observers ensured that large variations did not occur.

**Figure 1:**
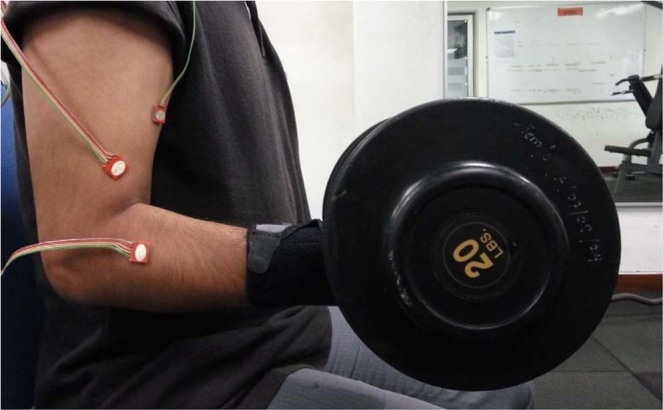
Posture for the flexion task.

**Figure 2:**
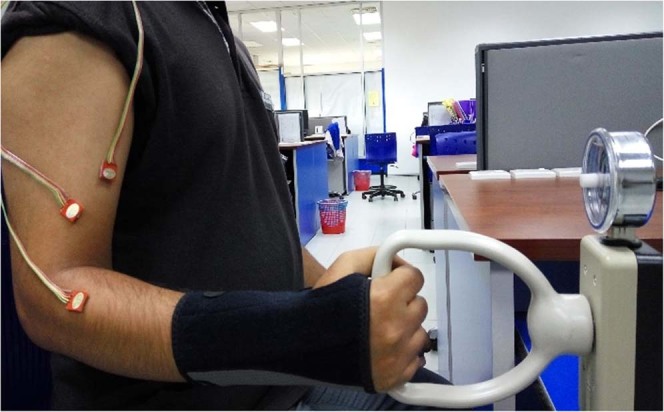
Posture for the pronation and supination tasks.

The subject’s MVC for the flexion task was determined as the maximum weight (dumbbell) during a sustained isometric hold for 3–4 seconds with the forearm in a supinated position (Fig. 1). Subsequently, for the pronation task with postural settings maintained (Fig. 2), the weights were replaced by a shovel handle of a wrist dynamometer (Baseline™ Evaluation Instruments, Fabrication Enterprises Inc., NY, USA) that was held with the wrist in neutral position. MVC for this task was considered as the maximum effort the subject could produce for 3–4 seconds during forearm pronation. Similarly, for the supination task using the same dynamometer, MVC was considered as the maximum effort the subject could produce for 3–4 seconds during forearm supination. Large variations (>±5%) in a trial were not allowed and considered unsuccessful. In addition, strong verbal encouragement was given to the subject to exert maximum effort with MVC being determined as the maximum from three successful trials.

In the second session of the experiment after a gap of 24–36 hours from the first session, the subject followed the same postural settings as adopted during the determination of MVC. The subject was then asked to perform isometric forearm flexion, pronation and supination tasks at 80% and 100% MVC for 6 seconds with a rest period of 2 minutes between any two consecutive trials and a rest period of 10 minutes between tasks. All trials were randomized in terms of tasks and MVC levels. During the trials, proper posture, off-axis precautionary measures, maximum ± 5% variations, announcement of time elapsed and verbal encouragement were observed. The generated signals were recorded in a personal computer for further analysis.

### Recording of MMG signals

MMG signals were collected using three accelerometers (ADXL335, Analogue Devices, USA; full-scale range = ±3 g; typical frequency response = 0.5–500 Hz; sensitivity = 330 mV/g; size = 15 mm × 15 mm × 1.5 mm; weight < 1.5 grams). The accelerometers were attached to the skin surface over the muscle bellies with double sided adhesive tape with the help of a physician present on site. The subject’s skin was appropriately cleaned and shaved prior to placement. It was assumed that the relative movement between the sensor and skin beneath is negligible. It was ensured that the arrangements used for sensor attachment over the skin do not interrupt the oscillations of the muscles and hence MMG signal recordings. The accelerometer axis transverse to the muscle fibres was used to record the MMG signals. The output from each of the sensor was connected to the data acquisition unit (NI cDAQ 9191 with the NI 9205 module, National Instruments, Austin, TX, USA), which was connected to a personal computer over Wi-Fi. The sampling rate was 1 kHz. MMG signals were recorded during all the trials, and each recording lasted 6 seconds. Data acquisition and storage were performed using custom-made programmes in LabVIEW™.

### Data analysis

**Figure 3:**
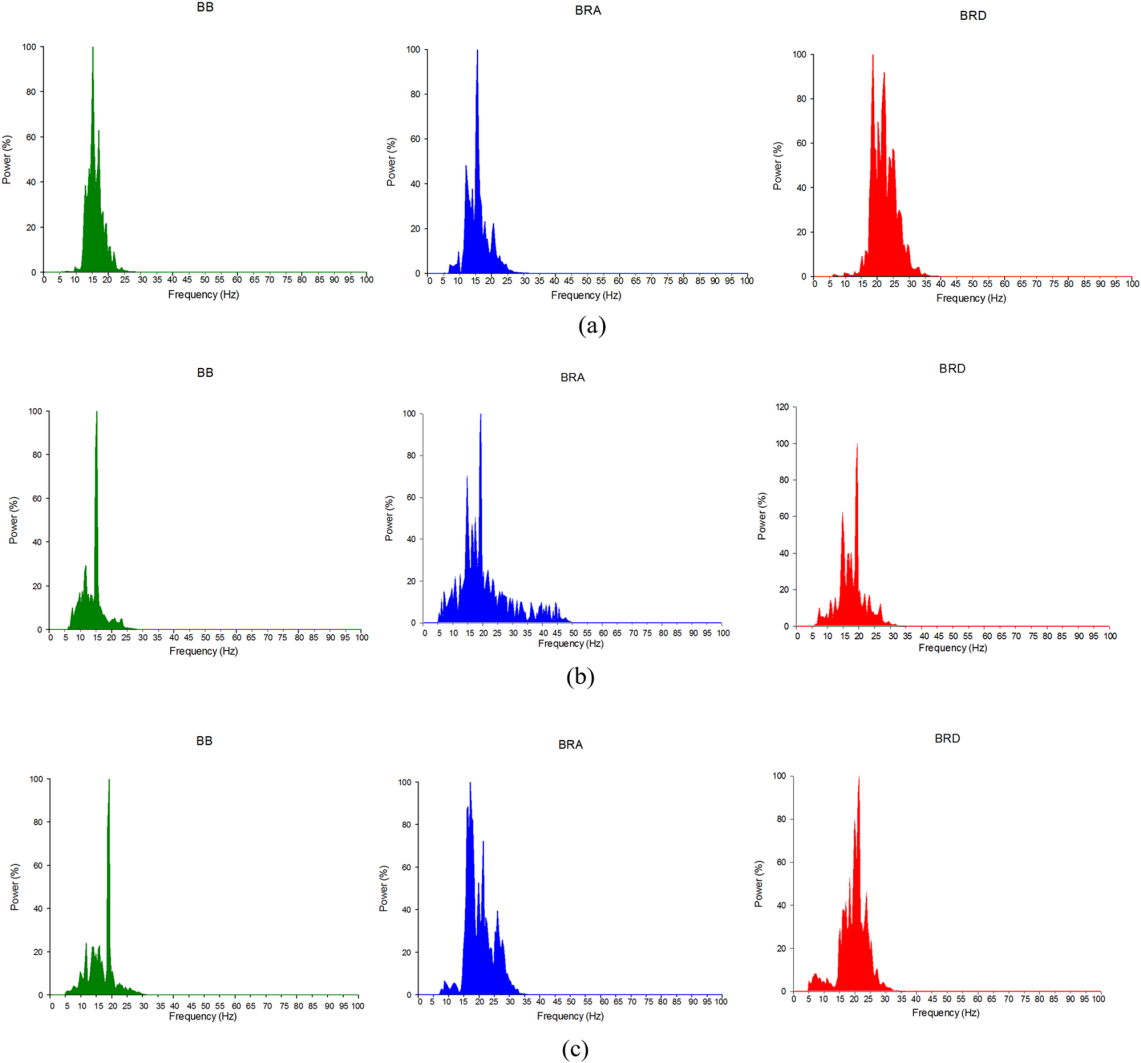
MMG signal power density spectrum of a subject performing forearm (**a**) flexion, (**b**) pronation and (**c**) supination tasks in BB, BRA and BRD muscles.

The data stored on a personal computer were first digitally bandpass filtered (fourth order Butterworth) at 5–100 Hz to obtain the MMG signals, and the MMG signals from the 2 seconds corresponding to the middle 33% of each 6 second isometric contraction were then extracted to remove the effect of signal transition, as recommended by^38^. The dominant power in MMG frequency spectrum was found outside the tremor range (5–12 Hz)^25^. The power density spectra of MMG signal from the three muscles of a subject performing forearm flexion, pronation and supination tasks are shown in Fig. 3. RMS was determined by taking the root mean square of the signal from each muscle and then normalized against the maximum RMS from the recorded MMG signal per MVC. Cross-correlation coefficients, which were calculated using Eq. (1), were employed to quantify CT in the MMG signals from two associated muscles^44^,

1 $${R}_{p,q}(\tau )=\frac{1}{a\times b\times \omega (\tau )}{\sum }_{n=0}^{N-1}{P}_{t}(n){Q}_{t}(n+\tau );\,1-N < \tau  < M$$

where *P*_t_ and *Q*_*t*_ are the MMG signals from the investigated muscle pair, $a=\sqrt{{\sum }_{n=0}^{N-1}{P}_{t}^{2}(n)}$, $b=\sqrt{{\sum }_{n=0}^{N-1}{Q}_{t}^{2}(n)}$, *ω* is a weighting factor, *M* and *N* are the lengths of *P*_*t*_ and *Q*_*t*_ respectively, and *τ* represents the time lag between the signals obtained at 1 − *N* to *M*. *R*_*p,q*_ (*τ*) was used to calculate the percentage of CT (or common signal) among the three muscle pairs – BB & BRA (P1), BRA & BRD (P2) and BB & BRD (P3).

### Statistical analysis

The statistical analyses were performed using IBM SPSS 20.0 (SPSS Inc., USA). Results [mean(SD)] of the Shapiro-Wilks’s test indicated that RMS [*p* < 0.05, skewness = 0.96(0.46)–3.60(0.46), kurtosis = 0.08(0.90)–15.67(0.90)], normalized RMS [*p* < 0.05, skewness = 0.60(0.46)–2.50(0.46), kurtosis = 0.83(0.90)–5.23(0.90)] and crosstalk [*p* < 0.05, skewness = 0.87(0.46)–2.67(0.46), kurtosis = 1.52(0.90)–7.08(0.90)] were found to be not normally distributed, while CA [*p* > 0.05, skewness = 0.21(0.46), kurtosis = 0.16(0.90)], LA [*p* > 0.05, skewness = −0.21(0.46), kurtosis = 0.16(0.90)], ST [*p* > 0.05, skewness = 0.58(0.46)–1.07(0.46), kurtosis = −0.05(0.90)–−0.54(0.90)] and ISD [*p* > 0.05, skewness = −0.00(0.46)–−0.00(0.46), kurtosis = −0.09(0.90)–−0.86(0.90)] were found to be normally distributed. As the data was found to be a mixture of both normal and not normally distributed parameters, hence non-parametric statistical tests were used for further analysis of the data. The intra-tester reliability of the four anthropometric parameters measured during the familiarization session and experimental procedures were checked using intra-class correlation co-efficient (ICC). The MMG data were not normally distributed, as demonstrated using a normality test, and non-parametric statistical tests were then used to further analyse the data. Statistical analyses using the Kruskal-Wallis test were performed to investigate the statistical differences among tasks and muscle pairs. Further post hoc analysis was performed using the Mann-Whitney tests to investigate the statistical differences between tasks and muscle pairs. The results of the statistical analysis were interpreted based on Cohen’s effect size (η^2^) as follows: <0.2 (small), >0.2–0.5 (medium) and >0.5 (high)^45^. Linear regression was performed to show the relationship of the anthropometric parameters of the arm with RMS and CT. Spearman’s correlation coefficients (*r*) at a significance level of 0.05 were used to describe the correlation between the variables as follows: 0.00–0.30 = negligible; ±0.30–±0.50 = low; ±0.50–±0.70 = moderate; ±0.70–±0.90 = high; ±0.90–±1.00 = very high^46^. The slope coefficient (*m*) for all correlations were obtained through linear regression analysis.

## Results

Table 2 presents the intra-tester reliability (ICC), typical error (TE) and minimum detectable change (MDC at 95% confidence level) values for the anthropometric parameters measured in the two sessions. The ICC values ranged from 0.972–0.999 and MDC ranged from 0.039–0.354. All the anthropometric measurements showed very high intra-tester reliability as ICC > 0.900.

**Table 2:** Intra-tester reliability of anthropometric parameters between familiarization session and experimental procedure.

No.	Anthropometric parameters	ICC (ICC_95%_)	TE	MDC
1	CA	0.998 (0.995–0.999)	0.127	0.354
2	LA	0.999 (0.997–0.999)	0.019	0.054
3	ST BB	0.992 (0.983–0.997)	0.045	0.125
4	ST BRA	0.996 (0.995–0.999)	0.041	0.115
5	ST BRD	0.993 (0.984–0.997)	0.045	0.125
6	ISD P1	0.996 (0.991–0.998)	0.127	0.352
7	ISD P2	0.972 (0.937–0.988)	0.014	0.039
8	ISD P3	0.994 (0.986–0.997)	0.056	0.156

*ICC: Intra-class correlation coefficient, ICC_95%_ = 95% lower and upper confidence intervals, TE = typical error calculated as SD of the difference between anthropometric measurements of two sessions divided by $\sqrt{2}$, MDC: minimum detectable change measured as ${\rm{TE}}\times 1.96\times \sqrt{2}$.

A summary of the statistical analyses of the normalized RMS values obtained for the muscles among the tasks, the values of CT obtained for the muscle pairs among the tasks and the values of CT obtained during the tasks among muscle pairs is provided in Table 3. The normalized RMS obtained for the BRA and BRD muscles showed significant differences among the three tasks with small effect size (*p* < 0.05, η^2^ = 0.141 – 0.153). Furthermore, the post hoc analysis revealed that the normalized RMS for pair ***a*** (flexion, pronation) was significant (*p* < 0.05) for the BRA and BRD muscles. With respect to CT, the P1 and P2 muscle pairs showed statistical significance among the three tasks with a small effect size (*p* < 0.05, η^2^ = 0.097 – 0.168). A post hoc analysis revealed that CT in pair ***c*** (flexion & supination) was significant (*p* < 0.05) for the P1 and P2 muscle pairs. In addition, the CT in the supination task showed statistical significance among the three muscle pairs with a small effect size (*p* < 0.05, η^2^ = 0.212). A post hoc analysis revealed that CT in pairs ***e*** (P2 and P3) and ***f*** (P1 and P3) was significant (*p* < 0.05) in all three tasks.

**Table 3:** Summary of statistical analyses [*p*-value (η^2^)] and details of the post hoc tests – ^a^(flexion, pronation), ^b^(pronation, supination), ^c^(flexion, supination), ^d^(Pair 1, Pair 2), ^e^(Pair 2, Pair 3), and ^f^(Pair 1, Pair 3).

Muscles	Normalized RMS among tasks	Muscle Pairs	CT among tasks	Tasks	CT among muscle pairs
BB	0.100 (0.041)	P1	**0.020 (0.097)** ^**c**^	Flexion	0.600 (0.011)
BRA	**0.020 (0.153)** ^**a,c**^	P2	**0.001 (0.168)** ^**c**^	Pronation	0.300 (0.027)
BRD	**0.004 (0.141)** ^**a**^	P3	0.500 (0.018)	Supination	**0.003 (0.212)** ^**e,f**^

*Bold font indicates statistical significance, *p* < 0.05.

**Figure 4:**
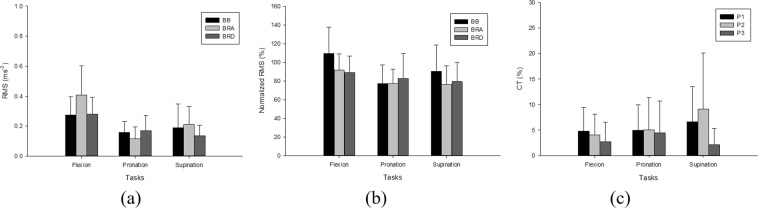
Mean(SD) values of (**a**) RMS (ms^−2^) (**b**) normalized RMS (%) and (**c**) CT (%).

Figure 4(a) shows the RMS mean(SD) values for the BB, BRA and BRD muscles during flexion, pronation and supination tasks. The overall range of the RMS values was 0.114–0.406 ms^−2^ in all three tasks. During flexion, pronation and supination, the RMS mean values ranged from 0.274–0.406 ms^−2^, 0.114–0.170 ms^−2^ and 0.136–0.189 ms^−2^ respectively. Figure 4(b) shows the normalized RMS mean(SD) values for all the muscles and tasks. The flexion task showed the highest values of the RMS and normalized RMS for all the muscles. Figure 4(c) shows the CT mean(SD) values for P1, P2 and P3 during the flexion, pronation and supination tasks. The overall range of the CT values was 2.163–9.145% in all three tasks. During flexion, pronation and supination, the CT values ranged from 2.748–4.810%, 4.484–5.062% and 2.163–9.145%, respectively. The flexion task showed the lowest CT values in all muscle pairs except P3.

Table 4 summarizes Spearman’s correlation coefficients (*r*) for RMS against the CA, LA and ST during all the tasks. The CA and LA showed negligible to low negative correlations with RMS, whereas the ST demonstrated negligible to moderate positive correlations. Most of the correlations with RMS did not show statistical significance (*p* > 0.05).

**Table 4:** Correlations (*r*) between anthropometric parameters and RMS.

Muscle Task	Muscle	RMS and CA	RMS and LA	RMS and ST
Flexion	BB	−0.435*	−0.135	0.299
	BRA	−0.461*	−0.043	0.225
	BRD	−0.155	−0.012	0.413*
Pronation	BB	−0.307	−0.148	0.467*
	BRA	−0.092	−0.032	0.379
	BRD	−0.076	−0.066	0.511*
Supination	BB	−0.225	−0.153	0.261
	BRA	−0.331	−0.089	0.276
	BRD	−0.243	−0.305	0.144

*Bold font indicates statistical significance, *p* < 0.05.

**Figure 5:**
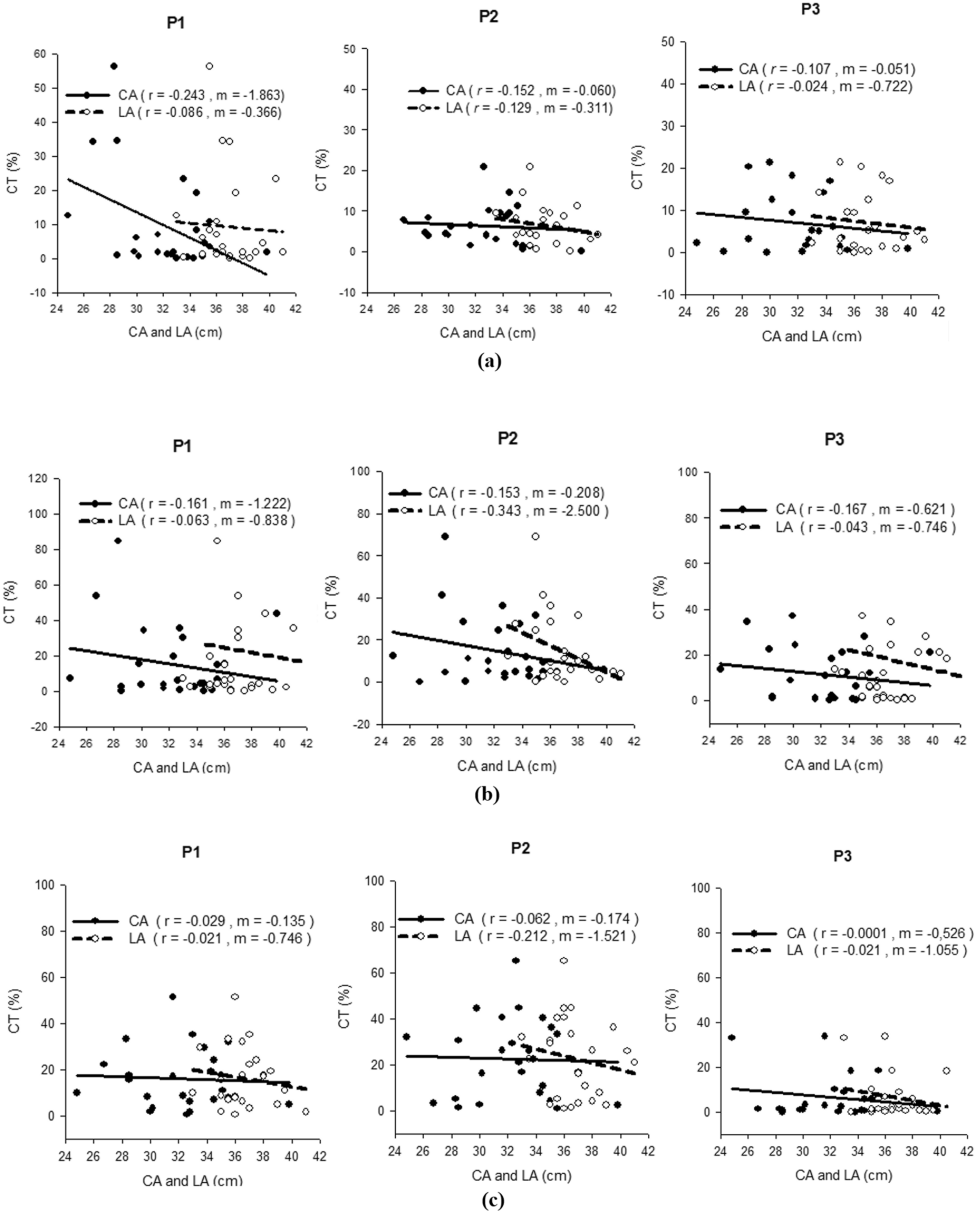
Correlations of CA and LA with CT for P1, P2 and P3 in the (**a**) flexion, (**b**) pronation, and (**c**) supination tasks.

Figure 5 demonstrates Spearman’s correlation coefficients (*r*) for CT with the CA and LA during all the tasks. Both parameters exhibited negligible to low negative correlations with CT in MMG signals. The *r* values for CA ranged from **−**0.0001–**−**0.243 in all three tasks. The CA exhibited *r* values during flexion, pronation and supination with ranges of **−**0.107–**−**0.243, **−**0.153–**−**0.167 and **−**0.0001–**−**0.062, respectively. The *r* value for LA ranged from **−**0.021–**−**0.343 in all three tasks. The LA exhibited *r* values during flexion, pronation and supination with ranges of **−**0.024–**−**0.129, **−**0.043–**−**0.343 and **−**0.021–**−**0.212, respectively.

**Figure 6:**
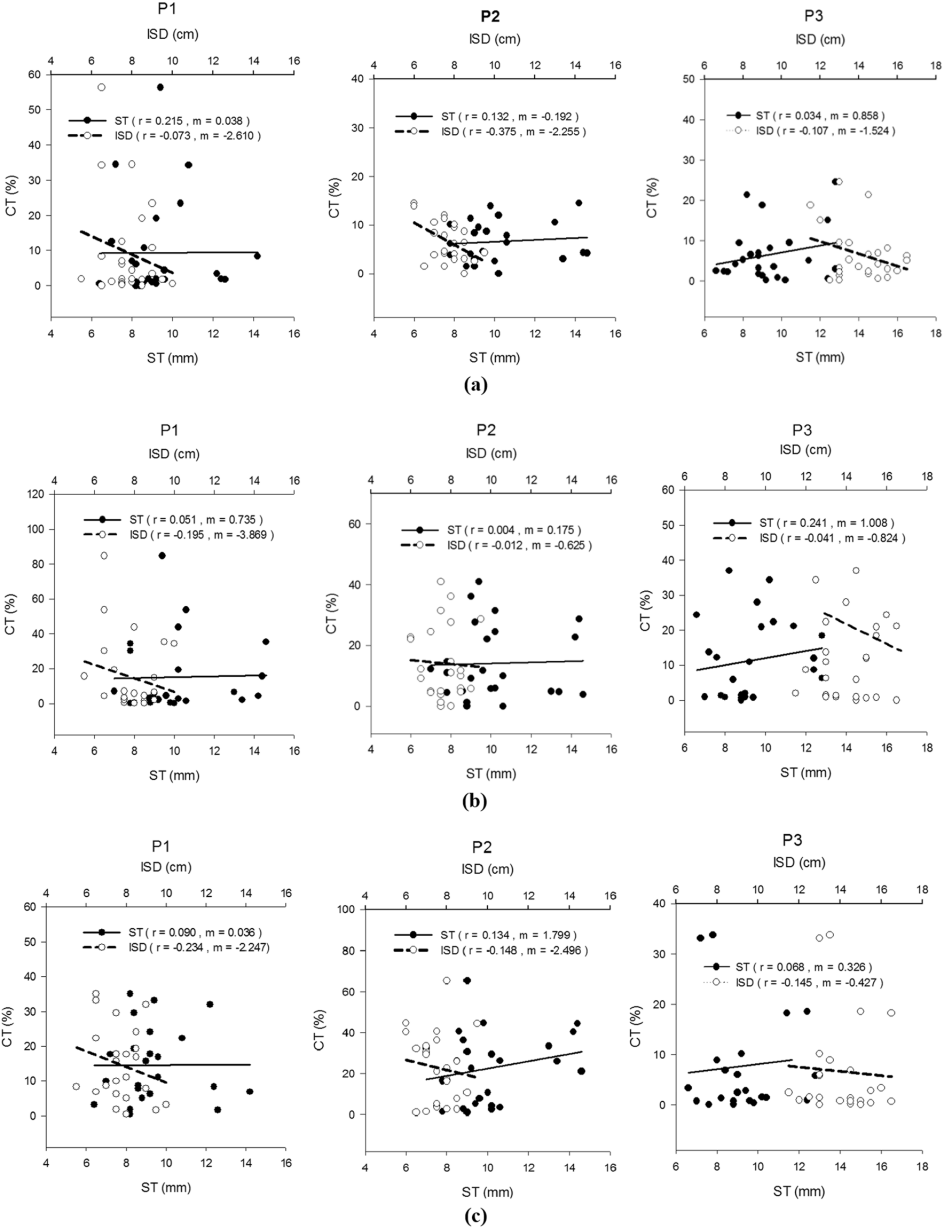
Correlations of ST and ISD with CT for P1, P2 and P3 in the (**a**) flexion, (**b**) pronation, and (**c**) supination tasks.

Figure 6 demonstrates Spearman’s correlation coefficients (*r*) for CT with the ST and ISD during all the tasks. The ST exhibited negligible to low positive correlations with CT in MMG signals, whereas the ISD showed negligible to low negative correlations. The *r* values for the ST ranged from 0.004–0.241 in all three tasks, and the *r* values for the ST during flexion, pronation and supination ranged from 0.034–0.215, 0.004–0.241 and 0.068–0.134, respectively. The *r* values for the ISD ranged from −0.012–−0.375 in all three tasks. The *r* values for the ISD during flexion, pronation and supination showed ranges of −0.073–−0.375, −0.012–−0.195 and −0.145–−0.234, respectively. All the correlations with CT did not show statistical significance (*p* > 0.05).

## Discussion

This study quantified the correlation of the RMS and CT of MMG signals recorded using an accelerometer with four anthropometric parameters, namely, the CA, LA, ISD and ST during elbow joint flexion, forearm pronation and supination tasks. Our initial findings revealed that the normalized RMS showed a significant difference among the three tasks in the BRA and BRD muscles (*p* < 0.05, η^2^ = 0.141–0.153) with small effect size. This finding is similar to that reported by^14^, who found that the RMS showed statistical significance in the forearm muscles among four wrist postures, and these observations were used further to characterize different wrist postures. In the current study, the value of CT among all three tasks showed a statistical significance (*p* < 0.05, η^2^ = 0.097–0.168) in two agonistic muscle pairs (P1 and P2) with a small effect size. In contrast^47^, observed statistical significance in CT in MMG signals among four wrist postures in an antagonistic muscle pair. Thus, the findings of the current study could be employed in the future for the characterization of forearm flexion, pronation and supination tasks even through the use of MMG signals from agonistic muscle pairs. The current study also investigated the mean(SD) values of the RMS, normalized RMS and CT during the three tasks. Normalized RMS was found to reflect the biomechanical activation of the muscles among the flexion, pronation and supination tasks for all three muscles, as shown in Fig. 4(b). The overall range of CT values between 2.163–9.145% as shown in Fig. 4(c), which while low, could potentially contribute to the trend of the RMS in the MMG signals from the elbow flexors as shown in Fig. 4(a).

Anthropometric variables were found to be correlated with muscle size by^23^, and the size of a muscle influences the parameters of MMG signals recorded using an accelerometer^2^. Hence, we believe that the correlation of the four anthropometric parameters with RMS and CT could provide additional evidence on the use and reliability of MMG as a muscle assessment tool. In the current study, a negligible to low negative correlation was found for CA with RMS and CT. This relationship is consistent with that found by^24^, who observed a low negative correlation between the two parameters. We found that the relationship between CA and CT is similar to that found in a previous study on sEMG, which observed that individuals with larger forearms exhibit decreased CT^26^. Interestingly, our findings are contrary to those reported by^25^, who observed a low positive correlation between forearm circumference and CT in MMG signals. This difference might be due to the fact that subjects with a similar musculature were recruited and included by^25^, which led to low variability in the forearm circumferences and might therefore have resulted in some bias in the results obtained. In contrast, the present study recruited subjects with a wider variation in musculature, as mentioned by^42^, resulting in variations in the arm circumference from 24–42 cm, as presented in Table 1. We also observed a negligible to low negative correlation for LA with both RMS and CT, similar to that reported by^25^, who found a low negative correlation between forearm length and CT. Although it has been established that RMS estimates the number of recruited motor units^48^, the findings of our study nevertheless reveal that the parameter is less affected by the arm length and circumference^24^ when measured using an accelerometer.

The current study observed a negligible to moderate positive correlation between RMS and ST, which is consistent with the results obtained by^24^ for the BB. A few studies^28–33^ have made some general observations on the ST and RMS from the quadriceps and found that these parameters could be related. Consequently, some other researchers^28–30^ have investigated and quantified the correlation between ST and RMS. These researchers found low positive correlations between the two parameters in normal subjects. The subjects considered in our study belong to second and third quartiles of BMI that cover a broad range of weight, height and length of arm of normal and healthy human beings, excluding malnutrition and obese subjects^43^. We note that the reported range of ST (6.8–14.8 mm) in our work is well within the accepted norms of the second and third quartiles of BMI^42^. Although the range of ST appears confined in our study, the limited associations observed between ST and RMS concurs with^28^ in which both normal and obese subjects were included (12% of significant low positive correlations) and in^24,29^ which also got limited associations (10% of significant low positive correlations and 0% of significant negligible positive correlations respectively) between the two parameters, with normal subjects. We note that the range of ST in our work is well within the range of ST investigated in those aforementioned works. With respect to CT and ST^34–36^, observed an increase in CT in sEMG signals with increases in the ST, but none of these studies quantified the correlation between these parameters. Furthermore, the observations reported by^35,36^ were based on simulated models, and the results were not validated on human subjects. To the best of our knowledge, this study constitutes the first attempt to quantify the correlation between ST and CT derived from MMG signals measured from the elbow flexor muscles using an accelerometer. Our results reveal negligible to moderate positive correlations between these parameters, and this trend concurs with those obtained in the aforementioned sEMG studies.

A negligible to low negative correlation was observed in this study between CT and the ISD, and this finding agrees with the previous studies that described CT in myographic signals as a function of muscle proximity^2^. For example, the closer proximity of the forearm muscles in comparison to that of the quadriceps muscles is thought to result in the higher mean CT values (69%) obtained by^37^ from for MMG signals compared with those (51%) found by^38^. The authors in^39^ observed that CT in sEMG signals from wrist-dedicated flexors was greater than that from digit-dedicated flexors, which could be due to the smaller ISD in the wrist- compared with the digit-dedicated flexors. Nevertheless, unlike sEMG, there is evidence showing that MMG signals are less effected by the sensor location over the muscle^8^. It is widely believed that MMG signals originate from the muscle, spread throughout the volume of the muscle and can be captured non-invasively using appropriate sensors^48^. Thus, the observed low correlations between the ISD and CT in our study provide further evidence showing that the protocol for MMG signal acquisition is invariant to sensor placement over the muscle, and this characteristic can be considered a benefit of MMG over other contemporary non-invasive techniques.

A point worth noting is, in this type of work the sample size becomes the ultimate determining factor for finding significant Spearman’s (*r*) associations between the investigated parameters. For example, a sample of *n* = 25 and α = 0.05 means that only *r* ≥ 0.337 could be found statistically significant. On the other hand, a sample size of *n* = 50 and α = 0.05 would have allowed *r* ≥ 0.235 to be significant, which if applied to RMS and ST, would have found all but 2 associations to be statistically significant in Table 4. While this may be true, the current study found that only 8% of all the correlations with RMS and CT were statistically significant (*p* < 0.05), and this proportion appears to be consistent with that found in a few other studies^27,29^ that also investigated and found low percentages of correlations between anthropometric variables and MMG signal parameters. Taken together, these findings suggest that RMS and CT might not be correlated with any of the four investigated anthropometric parameters but rather depend on the participation of specific muscles in an activity, the intensity of the activity, the muscle group, the muscle function (agonist or antagonist), the muscle fibre composition, the contractile properties of the muscle, and the non-contractile surrounding structures (bones or tendons). In addition, various other factors, such as the age of the subjects and the subject groupings (e.g., healthy or diseased, normal or obese, and trained or untrained), might also have an impact on the RMS and CT of MMG signals.

The present study nevertheless has a few limitations which if addressed could improve the robustness of the methodology used. The chosen anthropometric parameters have been used as surrogate estimates of skin thickness, subcutaneous fat thickness, muscle cross-sectional area, and muscle length in this study. As these anthropometric parameters are field measurements, they might exhibit larger errors of measurement than other, more precise techniques, such as magnetic resonance imaging, computed tomography, or ultrasound. The use of a modern technique to measure the anthropometric details of subjects in future can give more precise tissue measurements. Also, the use of ultrasound imaging of the concerned muscles during exercises can provide a better estimation of minor changes in the orientation of muscle fibers due to changes in the pennation angle, specifically during the pronation and supination exercises, while maintaining various elbow joint angles. Additionally, the identification and effect of variations in the filter between muscles, which comprises non-muscular structures, on crosstalk could be better understood using ultrasound imaging. Similarly, a study on the effect of variation in anthropometric variables at various levels of muscle effort on crosstalk levels in MMG signals might provide additional insights.

## Conclusion

CA, LA and ISD were found to exhibit negligible to low negative correlations with RMS and CT, whereas ST exhibited negligible to moderate positive correlations with RMS and CT. These findings suggest that the MMG signal parameters and CT acquired using accelerometers appear invariant to the four anthropometric parameters but might depend on other physiological and subject-based factors. The findings obtained in this study might aid our understanding of CT mechanisms in MMG signals, which could be useful in future research for devising techniques for the reduction or removal of CT in MMG.

## Data Availability

The datasets generated during and/or analyzed during the current study are available from the corresponding author on reasonable request.
